# Comparing Virtual Reality and Audio Analgesia for Reducing Dental Anxiety During Mandibular Third Molar Surgery in Adults: A Prospective Observational Study

**DOI:** 10.7759/cureus.101788

**Published:** 2026-01-18

**Authors:** Sagar R Dixit, Shreyas H Gupte, Janhavi Modi, Mihir M Naik, Purveet K Vasan, Riya S Rochlaney, Seema Gupta

**Affiliations:** 1 Department of Oral and Maxillofacial Surgery, Dr. G. D. Pol Foundation’s Y.M.T. Dental College and Hospital, Navi Mumbai, IND; 2 Department of Orthodontics, Kothiwal Dental College & Research Centre, Moradabad, IND

**Keywords:** anxiety, audio, extraction, third molar, virtual reality

## Abstract

Introduction

Dental anxiety frequently hinders treatment compliance, particularly during mandibular third molar surgery. Nonpharmacological distractions, such as virtual reality (VR) and audio analgesia, offer promising alternatives for managing anxiety and pain. This study compared the effectiveness of patient self-selected VR and audio analgesia versus no intervention in reducing anxiety and pain, physiological stress markers, and enhancing satisfaction during mandibular third molar surgery in adults.

Methods

In this prospective observational study, 150 patients aged 17-45 years undergoing impacted mandibular third molar extraction were self-selected into three groups (n = 50 each): VR (immersive calming environments via headset), audio analgesia (relaxing music via noise-canceling earbuds), and control (no distraction). The outcomes assessed included anxiety (Visual Facial Anxiety Scale), pain (Visual Analog Scale), heart rate and blood pressure, and satisfaction (5-point Likert scale) in the preoperative, intraoperative, and postoperative stages. The data were then statistically analyzed.

Results

The groups showed comparable baseline characteristics and preoperative anxiety (p > 0.05). Postoperatively, VR was associated with the lowest pain scores (median 2) and highest satisfaction (median 5), followed by audio analgesia (pain median 3-4; satisfaction 4) and control (pain median 5; satisfaction 3) (p < 0.001). VR correlated with higher heart rate and blood pressure, indicating possible arousal, and showed significant time-by-group interactions (p < 0.01).

Conclusions

Patient-preferred VR significantly reduced postoperative pain and increased satisfaction compared with audio analgesia or no intervention during mandibular third molar surgery. Despite higher heart rate and blood pressure in the VR group, subjective comfort was markedly improved. Audio analgesia provided moderate benefits as a simpler alternative. These findings support the use of VR as an effective, nonpharmacological tool to improve patient experience in adult oral surgery.

## Introduction

Dental anxiety poses a significant barrier to optimal oral healthcare and affects a substantial proportion of patients. Dental anxiety often arises from needle phobia, fear of procedural pain, and distressing sounds of dental equipment, such as handpieces, leading patients to avoid or delay treatment and compromising their oral health [1,2]. Needle phobia is especially prevalent, and it has been reported that 80% of adults in developed countries are apprehensive before their dental appointments, and 5% do not go to dentists because of fear and anxiety [3].

In oral and maxillofacial surgery, third molar extractions under local anesthesia are among the most common procedures; however, they frequently induce anxiety due to associated phobias. Although sedation is effective for highly anxious or uncooperative patients, it is resource-intensive and not suitable for all patients [4]. This has spurred interest in noninvasive, nonpharmacological alternatives, particularly digital technologies such as audio analgesia and virtual reality (VR), which distract patients from discomfort and anxiety [5,6].

Audio analgesia involves the delivery of calming sounds to create a soothing environment, influencing the limbic system to modulate emotional responses. It has shown promise in reducing anxiety, especially in pediatric patients, by engaging the auditory senses and mitigating perceptions of pain and fear [7]. However, a previous systematic review evaluating the effectiveness of music on preoperative pain and anxiety associated with third molar extractions concluded that music can reduce preoperative anxiety, but its effect on pain requires further investigation [8].

Conversely, VR offers immersive human-computer interaction through auditory, visual, and tactile stimuli, transporting users to a simulated world [5]. This diversion from the clinical setting effectively reduces pain and anxiety awareness. Although VR’s applications in dentistry have been explored more in children, its potential for adults undergoing third molar surgery merits further study [9].

This study aimed to compare the effectiveness of VR and audio analgesia in reducing dental anxiety during mandibular third molar surgery in adults. The primary objective of this study was to compare dental anxiety in patients using VR headsets or audio analgesia during mandibular wisdom tooth extraction. The secondary objectives included assessing heart rate, blood pressure, and patient satisfaction using a Likert scale across the VR, audio analgesia, and control groups.

## Materials and methods

This prospective observational study was conducted in the Department of Oral and Maxillofacial Surgery at Dr. G. D. Pol Foundation’s Y.M.T. Dental College and Hospital, Navi Mumbai, India, from June 2023 to May 2024. Unlike interventional trials, no randomization or assignment to interventions was performed; instead, participants were observed based on their self-selected preference for VR, audio analgesia, or no intervention. The study followed patients over time from enrollment through the surgical procedure and postoperative assessment to capture real-world outcomes in a natural clinical setting. All procedures were performed in a standard outpatient surgical operatory equipped with the necessary armamentarium and monitoring devices, ensuring compliance with institutional protocols for a sterile environment and patient safety.

The study included young and middle-aged adult patients aged 17-45 years who were scheduled for surgical extraction of impacted mandibular third molars. The target population consisted of individuals who met specific inclusion and exclusion criteria, reflecting a typical cohort presenting for such procedures in an urban dental setting in Brazil. Ethical clearance was obtained from the Institutional Ethical Committee prior to study initiation (YMTDC/IEC/2023/Th-023). Required permissions were secured from the concerned authorities, and written informed consent was obtained from all subjects, including details on data collection and observation of their chosen intervention (or lack thereof).

Based on an a priori analysis using G*Power software (version 3.1.9.2; Heinrich Heine University, Düsseldorf, Germany), a sample size of 50 participants per group was determined. This calculation assumed 80% statistical power, a 5% alpha error rate (two-tailed), and an anticipated effect size (minimal clinically important difference) of 0.25 for pain levels when comparing VR distraction to standard care during third molar extraction.

A convenience sampling technique was used to recruit consecutive eligible patients who presented to the department during the study period. This nonprobability approach was chosen to reflect real-world clinical practice, where patients self-select interventions based on their preferences or discussions with clinicians. Participants were divided into three groups based on their expressed preference prior to the procedure (50 in each Group A): Group A included patients who opted for VR intervention via a VR headset; Group B included patients who chose audio analgesia via wireless earbuds connected to a smartphone; and Group C included patients who preferred no intervention (control group). Group allocation was documented at enrollment, with no influence from the research team, to maintain the observational nature of the study. Eligible patients were identified during routine preoperative consultations. After explaining the study purpose and obtaining consent, participants were enrolled if they met the criteria and expressed a preference for one of the observation groups. No blinding was applied because this study aimed to capture naturalistic behaviors and outcomes.

The inclusion criteria encompassed patients classified as American Society of Anesthesiologists Class I [10], individuals aged 17 to 45 years, irrespective of sex, patients with no contraindications to the use of local anesthetic agents, and participants capable of understanding and following oral and written instructions. The exclusion criteria included immunocompromised patients, individuals with visual or auditory impairments, differently abled patients, and pregnant or lactating women.

Methods of measurement included assessment of anxiety using the Visual Facial Anxiety Scale (VFAS). No specific permission is required to use the VFAS from Cao et al. [11], as the article was published open access under the Creative Commons Attribution License (CC BY 4.0), which allows unrestricted use, distribution, and reproduction (including in research or clinical tools) with proper citation of the original source.

The VFAS is a validated tool with six facial expressions representing anxiety levels from “None” to “Highest” [11]. Pain was assessed using the Visual Analog Scale (VAS) [12], a 10 cm line where patients mark their pain intensity from “No Pain” (0) to “Worst Pain” (10). Patient satisfaction was evaluated using a 5-point Likert scale (typically ranging from 1 = “Not at all satisfied”/”Very dissatisfied” to 5 = “Very much satisfied”/”Very satisfied”) [13], and heart rate and blood pressure were monitored using a multipara patient monitor (GE Healthcare Dash 4000, GE Healthcare, Chicago, Illinois, USA). The generic 5-point Likert scale for patient satisfaction is in the public domain and free to use in research without requiring permission. The permission to use VAS has been obtained from John Wiley and Sons (license number 6183420977119).

The study instruments comprised a VR headset, specifically the Meta Quest 2 (model: Oculus Quest 2, Meta Platforms Inc., Menlo Park, California, USA), which provided immersive 360-degree videos of calming natural environments (such as beaches and forests) with a resolution of 1832 × 1920 pixels per eye and built-in audio; and audio analgesia delivered via a Samsung Galaxy S21 smartphone (Samsung Electronics, Suwon, South Korea) connected to Bose QuietComfort Earbuds (Bose Corp., Framingham, Massachusetts, USA), where patients listened to pre-selected relaxing instrumental music or nature sounds at a comfortable volume.

The surgical procedure began with Group A allocation based on preference, where patients self-selected into three groups: Group A opted for intervention using VR, Group B opted for audio analgesia, and Group C opted for no intervention. Preoperative preparation involved recording a detailed, complete case history for all patients, thoroughly explaining the procedure details, and obtaining informed consent for the surgical procedure. In the operative protocol, patients were taken to the surgical area, and scrubbing and draping were performed according to the standard sterile protocol. Intervention administration occurred pre-anesthesia: Group A patients wore the Meta Quest 2 VR headset and engaged with immersive content for distraction; Group B patients wore Bose QuietComfort Earbuds connected to a Samsung Galaxy S21 for audio playback; and Group C patients received no devices. Anesthesia and surgery followed, with local anesthesia (2% lignocaine hydrochloride with 1:80000 adrenaline) administered in all groups, and the surgical procedure was executed as per standardized surgical protocols, including incision, flap reflection, bone removal, tooth sectioning if necessary, extraction, and suturing.

The intervention protocol for VR involved placing a VR headset on the patient just before the injection of the local anesthetic agent, with the headset displaying a standardized VR video clip showing natural landscapes for 15 minutes. For audio analgesia, patients listened to standardized calming music using noise-canceling headphones for the same duration as the VR intervention. The outcome measures included the primary outcomes of dental anxiety assessed using the VFAS and pain perception measured using the VAS, as well as secondary outcomes of physiological parameters, including heart rate and blood pressure monitored using a multipara patient monitor, and patient satisfaction evaluated using a Likert scale. Preoperative evaluation consisted of anxiety using the VFAS scale and heart rate and blood pressure using a multipara patient monitor. Intraoperative evaluation included heart rate and blood pressure after 10 minutes of local anesthesia administration. Postoperative evaluation encompassed pain using the VAS scale, heart rate and blood pressure using a multipara patient monitor, and patient satisfaction using the Likert scale.

Data were managed using Excel (Microsoft Corporation, Redmond, WA, USA) and analyzed using IBM SPSS Statistics for Windows, Version 23.0 (Released 2015; IBM Corp., Armonk, NY, USA). Continuous variables, including heart rate, blood pressure, and pain scores, were assessed for normality using the Shapiro-Wilk test. All continuous variables, except for the pain and satisfaction scores, followed a normal distribution. Baseline (preoperative) characteristics were compared across the three groups using one-way ANOVA. For postoperative outcomes, data across preoperative, intraoperative, and postoperative time points were analyzed using mixed-model ANOVA to evaluate differences between groups and over time. Post hoc pairwise comparisons were conducted using Bonferroni correction. A p-value of <0.05 was considered statistically significant for all tests.

## Results

Comparison of baseline characteristics using one-way ANOVA (Table 1) revealed no statistically significant differences between the three groups prior to the intervention. The mean values for age (p = 0.323), preoperative heart rate (p = 0.962), systolic blood pressure (p = 0.809), and diastolic blood pressure (p = 0.833) were comparable, as indicated by p-values exceeding 0.05. This confirms that the groups were well-matched at the outset for these key demographic and physiological parameters. The absence of significant baseline differences ensured that any subsequent variations observed in postoperative outcomes could be more reliably attributed to the study interventions rather than to preexisting disparities between the groups.

**Table 1:** Comparison of baseline demographic and hemodynamic parameters among study groups Values are expressed as mean ± SD. One-way ANOVA was used for intergroup comparison: Group A: VR intervention using a head-mounted display during the procedure; Group B: Audio analgesia using wireless earbuds with calming music; Group C: Control group receiving no audiovisual intervention. p > 0.05 is considered nonstatistically significant. VR, virtual reality

Parameter	Group	95% CI for mean	Mean ± SD	F value	p-Value
Age (years)	A	28.60-31.04	29.82 ± 4.30	1.14	0.323
	B	27.64-29.80	28.72 ± 3.79		
	C	27.64-29.96	28.80 ± 4.09		
Heart rate (beats/min)	A	78.46-81.26	79.86 ± 4.93	0.04	0.962
	B	78.77-81.39	80.08 ± 4.61		
	C	78.28-81.36	79.82 ± 5.40		
Systolic blood pressure (mm Hg)	A	127.55-130.85	129.2 ± 5.79	0.21	0.809
	B	127.44-130.72	129.08 ± 5.77		
	C	128.04-131.56	129.80 ± 6.20		
Diastolic blood pressure (mm Hg)	A	78.79-81.85	80.32 ± 5.40	0.18	0.833
	B	78.37-81.35	79.86 ± 5.23		
	C	78.96-82.00	80.48 ± 5.36		

Mixed-model ANOVA (Table 2) revealed statistically significant main effects for time (pre-, intra-, and postoperative) and group, as well as a significant time-by-group interaction for systolic/diastolic blood pressure and heart rate (all p < 0.01). This indicates that all three parameters changed significantly across the perioperative period, the average values differed between the intervention groups, and crucially, the pattern of change over time was not the same for each group. Therefore, the study intervention had a differential impact on patients’ physiological trajectories.

**Table 2:** Mixed-model repeated-measures ANOVA showing effects of time, group, and interaction Repeated-measures mixed-model ANOVA was applied. Effect size reported as partial eta squared (η²). ^*^ p = 0.001 is considered statistically significant.

Source of variation	Systolic blood pressure			Diastolic blood pressure			Heart rate		
	F value	p-Value	Effect size	F value	p-Value	Effect size	F value	p-Value	Effect size
Time (preoperative, intraoperative, and postoperative)	95.79	0.001^*^	0.05	108.15	0.001^*^	0.07	176.36	0.001^*^	0.09
Group	8	0.001^*^	0.08	13.29	0.001^*^	0.12	6.64	0.002^*^	0.07
Time x group interaction	34.09	0.001^*^	0.03	42.55	0.001^*^	0.06	40.16	0.001^*^	0.04

Post hoc Bonferroni analysis (Table 3) clarified the group differences. For all parameters, Group C demonstrated significantly lower values than Group A (all p < 0.01). Differences between Groups A and B were significant for diastolic blood pressure (p = 0.013) and heart rate (p = 0.015), but not for systolic blood pressure. No statistically significant differences were found between Groups B and C. This indicates a distinct physiological response, with Group A consistently exhibiting the highest values and Group C the lowest, while Group B occupied an intermediate position that was not significantly different from that of Group C.

**Table 3:** Post hoc Bonferroni analysis for intergroup comparisons Bonferroni correction was applied for multiple comparisons: Group A: VR intervention using a head-mounted display during the procedure; Group B: Audio analgesia using wireless earbuds with calming music; Group C: Control group receiving no audiovisual intervention. ^*^ p < 0.05 is considered statistically significant. VR, virtual reality

Post hoc Bonferroni	Systolic blood pressure			Diastolic blood pressure			Heart rate		
	Mean difference (mm Hg)	t value	p-Value	Mean difference (mm Hg)	t value	p-Value	Mean difference (beats/min)	t value	p-value
A vs. B	-2.16	-2.03	0.131	-2.63	-2.89	0.013^*^	-2.67	-2.85	0.015^*^
A vs. C	-4.25	-4	0.001^*^	-4.68	-5.14	0.001^*^	-3.17	-3.39	0.003^*^
B vs. C	-2.09	-1.96	0.154	-2.05	-2.25	0.078	-0.5	-0.54	0.999

Analysis of nonnormally distributed outcomes using the Kruskal-Wallis test revealed significant differences in both postoperative pain and satisfaction levels across the groups (p = 0.001 for both). Post hoc comparisons showed an inverse relationship: Group A reported the lowest median pain score (2) and the highest satisfaction score (5), whereas Group C had the highest pain score (5) and the lowest satisfaction score (3). Group B scores were intermediate. This demonstrates that the interventions had a clinically meaningful impact, with the protocol for Group A being associated with the most favorable patient-reported outcomes of reduced pain and greater satisfaction (Table 4).

**Table 4:** Comparison of postoperative pain and patient satisfaction among groups Data are expressed as median and IQR (Q1-Q3). Intergroup comparisons were performed using the Kruskal-Wallis test. Group A: VR intervention using a head-mounted display during the procedure; Group B: Audio analgesia using wireless earbuds with calming music; Group C: Control group receiving no audiovisual intervention. Postoperative pain was assessed using the VAS, a validated 10-cm continuous scale ranging from 0 (no pain) to 10 (worst imaginable pain) [12]. Patient satisfaction was assessed using a 5-point Likert scale ranging from 1 (very dissatisfied) to 5 (very satisfied) [13]. ^*^ p < 0.05 is considered statistically significant. VAS, Visual Analog Scale; VR, virtual reality

Parameters	Group	Median	IQR	Test statistic (χ²)	p-value
Postoperative pain score	A	2	1-2	121.2	0.001^*^
	B	3	3-3		
	C	5	4-5		
Postoperative satisfaction score	A	5	4-5	76.24	0.001^*^
	B	4	3-4		
	C	3	3-3		

Analysis of preoperative anxiety levels using the chi-square test revealed no statistically significant difference in distribution across the three groups (p = 0.998). The proportions of patients in each anxiety category, from mild to severe, were closely comparable among Groups A, B, and C. This finding indicates that the groups were well-matched regarding baseline anxiety prior to the intervention. Consequently, the significant differences observed in physiological and patient-reported outcomes (pain and satisfaction) are unlikely to be confounded by preexisting disparities in anxiety levels among participants (Table 5).

**Table 5:** Comparison of anxiety levels across study groups Values are expressed as numbers and percentages. Comparison performed using the chi-square test: Group A: VR intervention using a head-mounted display during the procedure; Group B: Audio analgesia using wireless earbuds with calming music; Group C: Control group receiving no audiovisual intervention. Anxiety levels were assessed preoperatively using the VFAS, a validated categorical scale consisting of six facial expressions representing increasing anxiety levels from “none” to “highest” [11]. p > 0.05 is considered nonstatistically significant. VFAS, Visual Facial Anxiety Scale; VR, virtual reality

Anxiety level	Group A (n%)	Group B (n%)	Group C (n%)	χ² value	p-Value
	n (%)	n (%)	n (%)		
Mild	3 (6%)	4 (8%)	4 (8%)	1.01	0.998
Mild-moderate	3 (6%)	2 (4%)	3 (6%)		
Moderate	17 (34%)	18 (36%)	15 (30%)		
Moderate-high	16 (32%)	17 (34%)	17 (34%)		
Highest	11 (22%)	9 (18%)	11 (22%)		

## Discussion

The findings of this prospective observational study demonstrated that VR and audio analgesia, when self-selected by patients undergoing mandibular third molar surgery, differentially impacted physiological parameters, pain perception, and satisfaction. Group A (VR) exhibited the highest heart rate and blood pressure values, yet reported the lowest median pain scores and highest satisfaction. Group B (audio analgesia) occupied an intermediate position, with Group C (no intervention) showing the lowest physiological arousal, highest pain, and lowest satisfaction. These results suggest that nonpharmacological distractions can enhance patient-reported outcomes, although their effects on physiological markers may be more nuanced.

The superior performance of VR in reducing perceived pain and improving satisfaction aligns with the distraction theory, which posits that immersive multisensory stimuli compete for cognitive resources, thereby diminishing nociceptive processing [14]. VR’s ability to create a three-dimensional, interactive environment likely amplifies this effect by fostering a stronger sense of presence and diverting attention from procedural stressors, such as injections and drilling. This finding is consistent with those of prior randomized controlled trials (RCTs) conducted in dental settings. For instance, Yamashita et al. [15] reported significant anxiety reduction during impacted mandibular third molar extractions using VR under local anesthesia, attributing it to the immersive nature of the technology. Similarly, Mladenovic and Djordjevic [16] found that VR effectively lowered both anxiety and pain during third molar surgery, with patients experiencing less discomfort during anesthesia administration. A systematic review by Hao et al. [17] conducted a network meta-analysis of nonpharmacological interventions for tooth extractions, ranking relaxing music as the most effective for anxiety reduction, followed by VR. These studies provide mechanistic support: VR modulates the limbic system and prefrontal cortex, reducing emotional responses to pain, which may explain the lower VAS scores in Group A despite elevated physiological parameters in the latter.

The intermediate efficacy of audio analgesia in our study corroborates evidence from pediatric and adult dental literature, where auditory distraction mitigates anxiety by engaging the auditory cortex and altering emotional valence [18]. A meta-analysis by Klassen et al. [19] on music therapy during medical procedures, including dental extractions, showed moderate reductions in anxiety but inconsistent effects on pain. In the context of third molar surgery, a systematic review by Monteiro et al. [8] concluded that relaxing music reduces preoperative anxiety but requires further validation regarding its effect on intraoperative pain. Our findings extend this to adults, with Group B’s intermediate pain and satisfaction suggesting that audio is a viable, low-cost alternative, although less potent than VR due to its unisensory nature. The lack of significant differences between Groups B and C in some physiological measures may indicate that the anxiolytic effects of audio are subtler, relying on passive listening rather than active immersion.

An intriguing discrepancy in our results is the higher heart rate and blood pressure in the VR group despite favorable subjective outcomes. This could stem from the novelty or excitatory elements of VR, such as dynamic visuals, leading to transient sympathetic activation without increasing perceived distress, a phenomenon noted in some VR studies as “arousal mismatch” [20]. However, Furman et al. [21] observed lower blood pressure and pulse in VR-exposed patients during dental procedures. Alternatively, self-selection bias in our observational design might have played a role; patients opting for VR may have had unmeasured traits, such as higher baseline arousal tolerance. However, the absence of baseline differences mitigated this concern. Mixed-model ANOVA confirmed time-by-group interactions, indicating that the interventions modulated dynamic physiological responses.

Clinically, these results imply that VR can be integrated into routine oral surgery protocols for adults with dental anxiety, potentially improving compliance and reducing reliance on sedatives. Given its noninvasive nature, VR may enhance patient-centered care in outpatient settings, particularly for third molar extractions, which are prone to phobia-induced avoidance. Audio analgesia offers a simpler and more accessible option for resource-limited practices. Implementing these measures could lower overall healthcare costs by minimizing procedure interruptions and postoperative complications.

Limitations include the observational design, which precludes causal inference due to potential confounding from self-selection; patients with higher anxiety might prefer VR, although baseline equivalence suggests minimal bias. The single-center setting limits generalizability, and the lack of long-term follow-up overlooks the sustained effects. Future RCTs with larger, diverse samples and objective anxiety biomarkers (such as cortisol) are warranted to confirm these findings.

## Conclusions

This prospective observational study revealed that VR, when self-selected by patients, is associated with significantly lower postoperative pain perception and higher patient satisfaction during mandibular third molar surgery than audio analgesia or no intervention. Despite elevated physiological parameters (heart rate and blood pressure) in the VR group, subjective outcomes were markedly superior, highlighting VR’s potent distraction effect. Audio analgesia offers intermediate benefits, supporting its role as a simple alternative. These nonpharmacological interventions have the potential to enhance the patient experience and reduce anxiety-related barriers in adults undergoing oral surgery, warranting broader clinical adoption.
